# Conditional ablation of TGF-β signaling inhibits tumor progression and invasion in an induced mouse bladder cancer model

**DOI:** 10.1038/srep29479

**Published:** 2016-07-05

**Authors:** Yu Liang, Fengyu Zhu, Haojie Zhang, Demeng Chen, Xiuhong Zhang, Qian Gao, Yang Li

**Affiliations:** 1 Department of biology, School of Life Science, Anhui Medical University, Hefei, Anhui 230031, China; 2 Department of Urology, Huadong Hospital, Fudan University. Shanghai, 200040, China; 3 Department of biology, Case western reserve university, 2080 Adelbert Road Cleveland, OH 44106, United States

## Abstract

The role of transforming growth factor-β (TGF-β) signaling in cancer progression is still under debate. To determine the function of TGF-β signaling in bladder cancer progression, we conditionally knocked out the Tgfbr2 in mouse model after a N-butyl-N-4-hydroxybutyl Nitrosamine induced bladder carcinogenesis. We found the ablation of TGF-β signaling could inhibit the cancer cell proliferation, cancer stem cell population and EMT, hence suppressed the invasive cancer progression, which is similar with the result of TGF-β receptor I inhibitor treatment. These findings recognize the roles and mechanisms of TGF-β signaling in bladder cancer progression *in vivo* for the first time.

Bladder cancer (BCa) is the most common malignancy in urinary tract, it is the 6^th^ common and the 9^th^ cause of deaths of all male malignancies worldwide^1^. In 2015, 32,900 people died of BCa and the estimated new cases were 80,500 in China^2^. Pathological studies indicate that BCa comprises two major groups^3^: superficial carcinoma usually recurs, but rarely progresses^4^,^5^, while muscle-invasive bladder cancer is more aggressive with worse prognosis^6^. Despite the progress we have made in recent years, especially the insights of genome-wide landscape in BCa^7^, our understanding of its tumorigenesis and progression is still limited.

Transforming growth factor-β (TGF-β) family encodes pro-fibrotic growth factors which are involved in many physiological processes such as wound healing and tissue fibrosis by inducing fibroblast differentiation to myofibroblasts^8^, it has been proved to play pivotal roles in cell migration, survival, proliferation, and differentiation^9^. The signaling is initiated with ligand-induced oligomerization of TGF-β receptor1/2 complex (serine/threonine kinase) and phosphorylation of the cytoplasmic Smad2 and Smad3, which results in their translocation to the nucleus along with the common signaling transducer Smad4. Activated Smads will promote the transcription of multiple downstream targets by coordinating with other transcriptional factors such like AP-1, RUNX, *et al*.^10^.

TGF-β signaling is well known for its contribution to cancer development by promoting invasiveness and metastasis and inducing the epithelial-to-mesenchymal transition (EMT)^11^. However, the opinions of whether TGF-β signaling could activate or suppress the tumor growth is still controversial, possibly due to the cancer context and research model used^12^. As for the BCa, TGF-β signaling is indicated to participate in its tumorigenesis^13^ and promote EMT^14^. However, there is still no *in vivo* data characterizing its role in the BCa. In the current study, we used the mouse model of KRT5-Cre driven conditional knockout of TGF-β2 and N-butyl-N-4-hydroxybutyl Nitrosamine (BBN) induced BCa to demonstrate that ablation of TGF-β signaling could inhibit the progression, invasion and cancer stem cell population of BCa as well as the EMT. Treatment of TGF-β receptor1 specific inhibitor, LY364947, after the tumor transformation, also inhibited BCa tumor growth. To our knowledge, these findings provided the first *in vivo* evidence for the crucial role of TGF-β signaling in bladder cancer progression and unveiled the possible mechanism of TGF-β mediated tumor growth and invasion.

## Results

### BBN-induced bladder carcinogenesis

**Figure 1:**
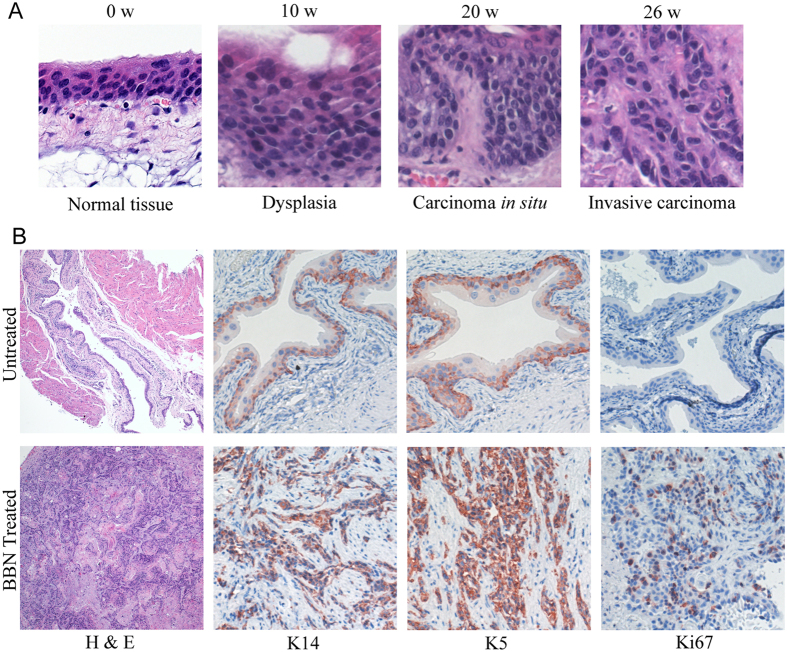
Histopathology of BBN-induced bladder carcinoma in mouse model mimics progression of human urothelial CIS to invasive carcinoma. (**A**) Histopathological analysis (H&E staining) of BBN-induced bladder carcinogenesis at indicated time and stages with typical morphology changes. (**B**) Immunohistochemical staining of tumor tissues from BBN or vehicle treated groups were fixed and immunostained by K5, k14 and Ki-67 antibodies, respectively.

To investigate the role of TGF-β signaling in bladder cancer progression *in vivo*, we made use of a BBN-induced bladder carcinogenesis mouse model that shares molecular similarities to human disease^15^. Only male mice with correct genotype were selected for the study since the female mice are more resistant to BBN treatment^16^. Bladders of the BBN treated mice developed progressively from dysplasia and carcinoma *in situ* to invasive carcinoma (Fig. 1A). Compared with the vehicle treated group, all mice (8/8) exposed to BBN for 26 weeks were observed to develop muscle-invasive bladder cancers, which were confirmed by H&E staining in histologic sections showing invasion into muscle layers (Fig. 1A). Previously studies have demonstrated that keratin5 (K5)-expressing basal cells are main progenitors of carcinoma *in situ* and invasive carcinoma in bladder by lineage-tracing experiments^17^. Immunohistochemistry results showed that a large portion of the invasive carcinoma induced by BBN were keratin14 (K14) and K5 positive (Fig. 1B). In addition, indicated by Ki-67 staining, the invasive tumor cells are highly proliferating, while most normal urothelial cells are postmitotic (Fig. 1B).

### TGF-β signaling is required for BBN-induced invasive bladder cancer progression

**Figure 2:**
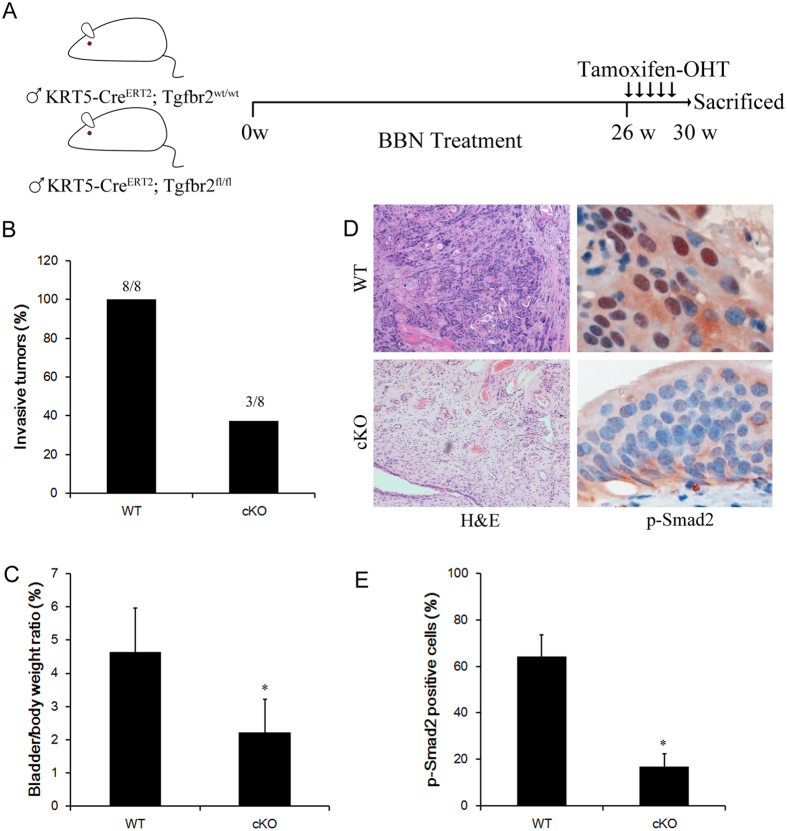
Genetic ablation of TGF-β signaling suppress bladder cancer progression. (**A**) Schematic diagrams show the experimental strategies. Tamoxifen was injected into KRT5-CreER;Tgfbr2^flox/flox^ or wild type control mice on 5 consecutive days after 26 weeks of BBN exposure to achieve maximal frequency of floxp-mediated cell ablation. 4 weeks after the Tamoxifen injection, mice were sacrificed and bladders samples were analyzed by immunostaining. (**B**) Conditional Tgfbr2 ablation reduced the incidence of invasive bladder cancers development in Tgfbr2 knockout (cKO) mice relative to the wild type (WT) mice. Statistical analysis was performed by student’s t-test. WT(n = 8), cKO(n = 8). (**C**) G/B ratio was calculated as (gross bladder weight/body weight) × 100% and analyzed with the one-way AVOVA for the different groups. (**D**) Representative sections from the bladders of WT or cKO mice were stained for phosphorylated Smad2 antibody. **E**, Quantification of the phosphor-Smad2 positive cells ratio in the indicated sections *P < 0.05.

TGF-β signaling is well-known for its involvement in cancer invasion^11^, to examine the role of TGF-β pathway in bladder cancer invasion *in vivo*, we crossed *Tgfbr2 flox* mice with *K5*^*Cre-ER*^ mice, and tamoxifen was applied to tumor-bearing mice after 26 week of BBN treatment. Four weeks after Tamoxifen administration, tumors from both control and conditional *Tgfbr2* knockout mice were collected for further analysis (Fig. 2A). All control mice developed invasive bladder cancers, while only 37.5% of conditional knockout mice developed invasive bladder cancer, as confirmed by H&E staining (Fig. 2B,D). As a function of cancer progression, relative gross bladder/body weight ratio was calculated to estimate the tumor growth in mice of bladder cancer model. The average ratio of the knockout group was 2.23% ± 1.00%, which was significantly lower than that of the control group(4.65% ± 1.31%) (P = 0.022). Immunohistochemistry analysis revealed strong phosphorylation of Smad2 of Ser465/467, which is an activation marker of TGF signaling^18^, in control invasive cancer cells. In contrast, Tamoxifen administration to *K5Cre-ER; Tgfbr2*^*flox/flox*^ mice led to significant decrease of Smad2 phosphorylation in tumors (Fig. 2D,E).

### Blockage of TGF-β signaling led to decrease of cancer stem cell population

**Figure 3:**
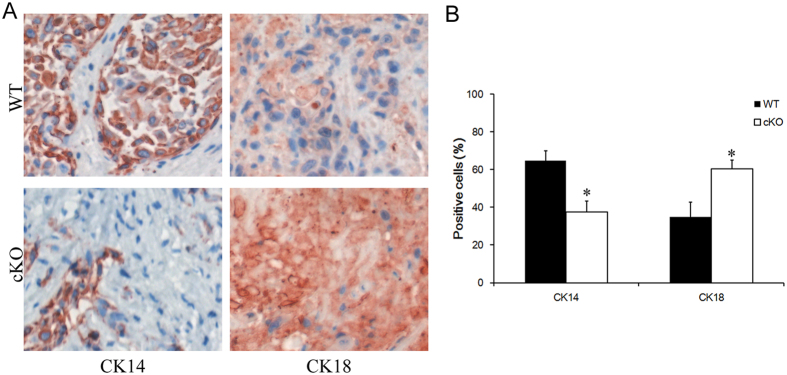
TGF-β signaling blockage decreased the bladder cancer stem cell population. (**A**) Immuohistochemical analysis of bladders cancer samples from wild type (control) or Tgfbr2 knockout (cKO) mice treated with BBN by K14 and K18 antibodies. (**B**) Quantifications of K14^+^ and K18^+^ cells percentage in bladder cancer samples after BBN treatment. Data represent mean ± SEM. *p < 0.05; n = 8; Student’s t-test.

K14-expressing tumor cells have been previously identified as cancer stem cells population in bladder cancer^19^. We reasoned that deletion of *Tgfbr2* may cause a decrease of K14^+^ cancer stem cells population, which subsequently impaired the invasive capability of tumor. Our data displayed a noticeable decrease of K14 expression in conditional *Tgfbr2* knockout tumors relative to the normal control (Fig. 3A,B). In addition, K5 showed similar expression pattern with K14 (data not shown). Consistently, we detected a substantially increase of Keratin18 (K18), a differentiated tumor cell marker, in *Tgfbr2* conditional knockout tumors (Fig. 3A,B). In invasive bladder tumors from wild-type mice, there were 65.4% K14^+^ tumor cells and 34.6% K18^+^ differentiated cells. However, in the tumors from *Tgfbr2* conditional knockout mice, these cell population ratio were considerably altered to 16.3%(K14^+^) and 83.7%(K18^+^), respectively (Fig. 2C). The frequency of these cellular compartments confirmed a functional role of TGF-β signaling in regulating cancer stem cell population.

### TGF-β signaling affects BBN-induced tumor proliferation and apoptosis

**Figure 4:**
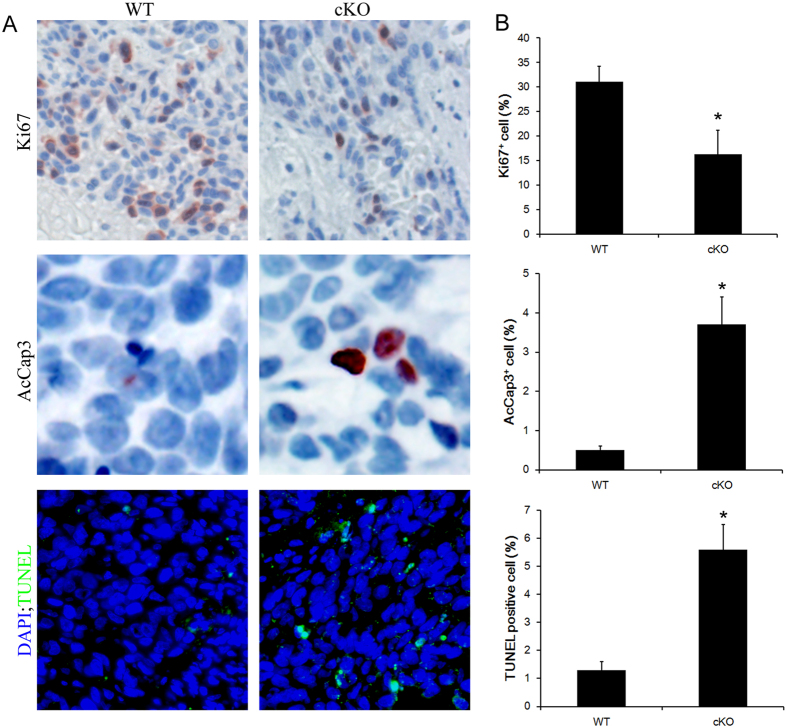
Influence of TGF-β signaling on bladder cancer proliferation and apoptosis. (**A**) Immuohistochemical analysis of bladders cancer samples from wild type (WT) or Tgfbr2 knockout (cKO) mice treated with BBN by Ki-67, active-caspase3(AcCap3) antibodies as well as stained by TUNEL assay. (**B**) Quantifications of Ki-67^+^, AcCap3^+^ and TUNEL positive stained cells percentage in bladder cancer samples after BBN treatment. Data represent mean ± SEM. *p < 0.05; n = 8; Student’s t-test.

To examine whether TGF-β signaling can influence bladder cancer proliferation and apoptosis *in vivo*, we assessed cell proliferation and apoptotic cell death by immunostaining for Ki67, active-caspase3 and TUNEL assay, respectively, in the BBN-induced bladder tumors harvested from mice mentioned above. The proliferation index, defined as the percentages of Ki67^+^ in tumors, was statistically different between control and *Tgfbr2* conditional knockout groups (Fig. 4A,B) (proliferation index: 31.5 ± 6.2 versus 17.1 ± 4.2, respectively). We also detected significant increase of apoptotic cells percentage in *Tgfbr2* conditional knockout tumors compared with control tumors (apoptotic index: determined by AcCap3: 0.5 ± 0.1 versus 3.7 ± 1.8, and by TUNEL: 1.3 ± 0.3 versus 5.6 ± 0.9, respectively) (Fig. 4A,B). Thus, these findings indicated that the TGF-β signaling contributes to bladder cancer cell proliferation and apoptosis in BBN-induced bladder cancer and support the hypothesis that TGF-β signaling promotes bladder cancer progression.

### TGF-β signaling affects epithelial–mesenchymal transition (EMT) of tumor cells

**Figure 5:**
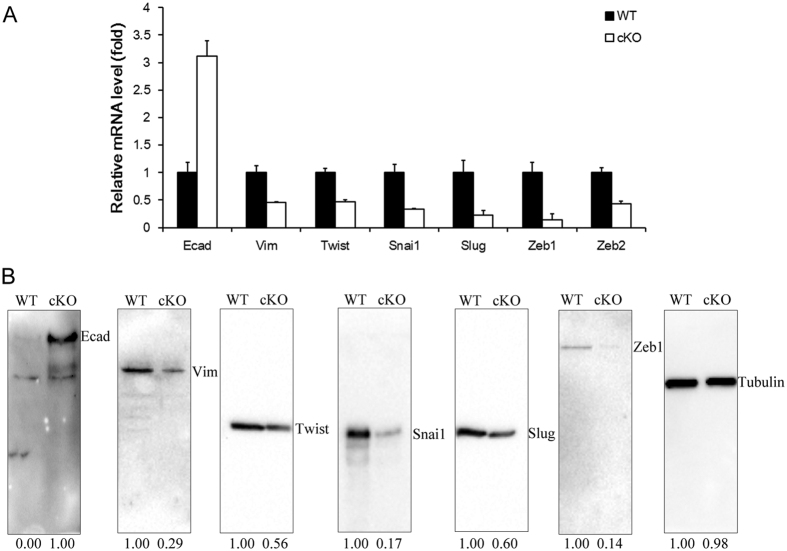
Ablation of TGF-β signaling regulated bladder cancer EMT. (**A**) Quantitative real-time PCR analysis of the epithelial and mesenchymal markers mRNA level in wild type (WT) or Tgfbr2 knockout (cKO) mice. (**B**) Protein levels of the same set of markers analyzed by Western blot.

TGF-β receptor signaling plays an essential role in promoting EMT and modulating the migration and invasion of cancer cells^20^. Here we collected tumor samples from control and *Tgfbr2* conditional knockout mice and analyzed the expression of a number of EMT marker genes. Following Tamoxifen treatment, the mRNA expression levels of the mesenchymal markers, including *Vimentin, Slug, Snai1, Twist, Zeb1* were downregulated in conditional knockout tumors (Fig. 5A). Conversely, the mRNA expression of the epithelial marker *E-cadherin* was upregulated (Fig. 5A). Consistently, Western blot results confirmed that protein levels of Vimentin, Slug, Snai1, Twist, Zeb1 were reduced in conditional knockout tumors (Fig. 5B). While augment expression of E-cadherin protein was detected in conditional knockout tumors as compared with control tumors (Fig. 5B). Our data revealed that TGF-β receptor signaling regulated genes involved in EMT.

### Small molecular inhibitor of TGF-β signaling inhibited BBN-induced tumor invasion

**Figure 6:**
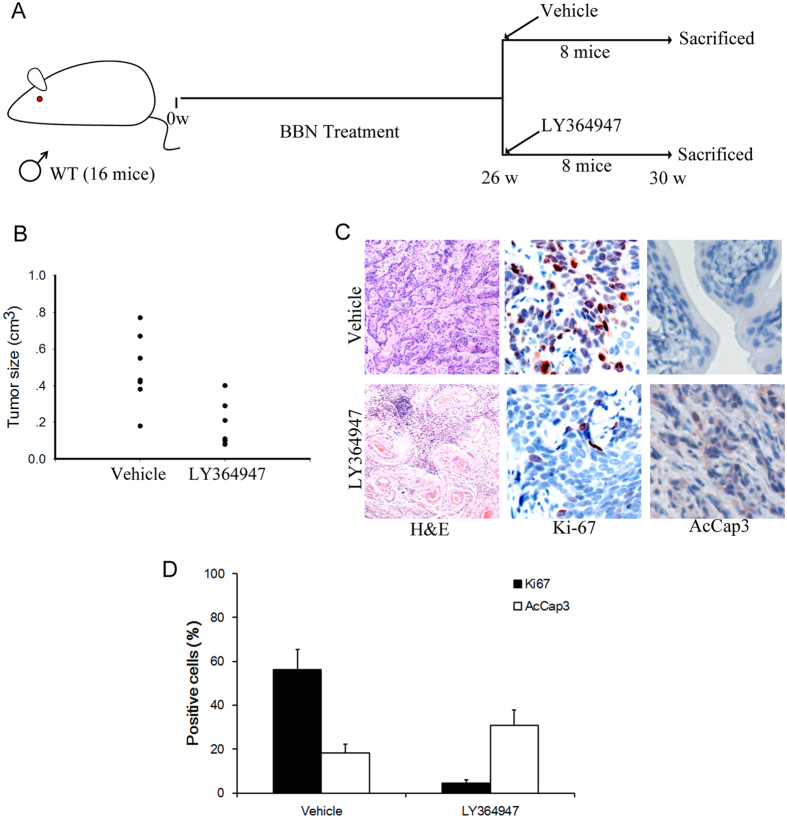
TGF-β signaling inhibitor reduced BBN-induced tumor invasion. (**A**) Schematic diagrams show the experimental strategies. 8 Balb/c mice were administrated with LY364947 or vehicle after 26 weeks of BBN exposure, respectively. 4 weeks after the LY364947 injection, mice were sacrificed and bladders samples were analyzed by immunostaining. (**B**) Quantification of tumor lesion size from different treatment groups. Statistical analysis was performed by Student’s t-test. *p < 0,05; n = 8. (**C**) H&E staining and immuohistochemical analysis of bladders cancer samples from LY364947 or vehicle injected mice with BBN exposure by Ki-67 and active-caspase3 (AcCap3) antibodies. (**D**) Quantification of the Ki67 and Accap3 positive cells ratio in the indicated sections *P < 0.05.

To test whether inhibition of endogenous TGF-β signaling by specific small molecule inhibitors can restrain the invasion and progression of bladder cancer, 16 male Balb/c mice with 26 weeks of BBN treatment were injected intraperitoneally with LY364947^21^ (1 mg/kg) 3 times a week for 4 weeks or PBS, respectively (8 mice per group, Fig. 6A). As expected, bladder cancers were sensitive to LY364947, which significantly inhibited tumor formation and growth induced by BBN in mice (Fig. 6B). In tissue sections from LY364947-treated bladder cancer, considerably more activated caspase-3 positive nuclei were demonstrated than in sections from vehicle treated cancers (Fig. 6C,D). On the other hand, the number of proliferating (Ki-67^+^) cells was dramatically diminished in the tumors treated with LY364947 (Fig. 6C,D). Thus, TGF-β signal inhibitors like LY364947 may present an effective therapeutic avenue for bladder cancer.

## Discussion

Genetic alterations, as well as epigenetic gene regulation, in various signaling pathways are involved cancer development^22^,^23^,^24^,^25^. The TGF-β signaling pathway is involved in various aspects of psychological and physiological processes, including genesis and progression of urinary bladder cancer^26^,^27^. It has been shown that TGF-β1 secretion levels correlates with more aggressive phenotype of bladder cancer cell lines^28^. Moreover, knockdown of TGFβRI by siRNA significantly reduced invasiveness of bladder cancer cells^29^. In the present study, we set off to define the role of TGF-β signaling pathway in bladder cancer invasion *in vivo*. We showed that depletion of *Tgfbr2* reduced the invasiveness of bladder cancer induced by BBN. Moreover, chemical inhibitor of TGF-β signaling also diminished bladder cancer progression. Therefore, TGF-β signaling pathway components can serve as promising candidates for the development of novel therapeutic strategies for anti-bladder cancer treatment. A series clinical trials of TGF-β inhibitors on different types of cancer have been launched so far, which mainly aim to influences tumor microenvironment by inhibiting fibrosis, angiogenesis, metastasis, and activating immune-related host response^30^. Among which a TGFbR/ALK1 inhibitor PF-03446962 has been approved for phase II trial of bladder cancer treatment^31^.

TGF-β signaling exerts its pro-proliferating and anti-apoptotic effects through activated either Smad proteins or the extracellular signal regulated kinases 1 and 2 (ERK1/ERK2)^32^. In *Tgfbr2* conditional knockout mice, we observed downregulation of p-Smad2 in tumor cells, which correlated with increase of tumor cell apoptosis and increase of proliferation activity. In addition, we made the novel observation that LY364947, a Tgfbr1 inhibitor, could also inhibit cell survival and proliferation in BBN-induced carcinogenesis. Hence, TGF-β signaling plays a key role in cell survival for supporting the invasive and metastatic process of bladder cancer.

Long-lived cancer stem cells are the roots of cancer, which are able to self-renew and differentiate, initiate and propagate cancers^33^. Study has shown that TGF-β-activated cells are the cancer stem cell population in skin squamous cell carcinoma. These cells display slow-cycle and chemoresistant properties^34^. Although whether TGF-β-activated are also cancer stem cells in bladder cancer remains closer investigation, our results revealed that ablation of TGF-β signaling led to decrease of K14^+^ cancer stem cells population, which might be responsible for the decrease of tumor progression and invasion.

TGF-β can also promote tumor invasion and metastasis by inducing an EMT. Epithelial tumor cells undergoing EMT become more invasive, resulting in dissemination of cancer cells to metastatic location from the carcinoma *in situ*. Essentially, expression of all known EMT transcription factors, including Snail, ZEB and bHLH families, is activated by TGF-β, either through a Smad-dependent mechanism or indirectly through activation of other transcription factors or relief of repression^35^,^36^,^37^. How TGF-β regulates EMT in bladder cancer is not completely understood.

In conclusion, this study demonstrated that genetic deletion of Tgfbr2 or treatment of Tgfbr1 small molecule inhibitor LY364947 led to decrease of bladder tumor invasion and progression. Therefore, the results obtained in this study utilizing TGF-β signaling antagonists provide a rationale for further pre-clinical studies in order to dissect the TGF-β signaling pathway to obtain full benefit from this pathway targeted therapy as a single agent or in combination against bladder cancer as well as other solid tumors dependent upon TGF-β signaling.

## Materials and Methods

### Mice model

KRT5-Cre^ERT2^ and Tgfbr2^*flox/flox*^ mice were obtained from Jackson Laboratory, Balb/c male mice were provided by Shanghai Laboratory Animals Center (SLAC). BBN (Santa Cruz) was dissolved in drinking water at a concentration of 0.05% (w/v) and provided to transgenic or wild type male mice for 30 weeks as previous described^38^. Tamoxifen (T5648) were purchased from Sigma, BBN(sc-486264) and LY 364947 (sc-203122) were obtained from Santa Cruz. All animal procedures were performed under a protocol approved by the Laboratory Animal Center of Anhui Medical University and in accordance with National Institutes of Health guide for the care and use of Laboratory animals (NIH Publications No. 8023, revised 1978).

### Immunohistochemistry

Paraffin sections of bladder cancer tissue samples from mice were antigen retrieved, blocked and processed as described before^26^,^39^. Primary antibodies of mouse Krt-5 (MA5-17057), Ki-67(PA5-19462) and Smad2 pSer465/pSer467 (44-244G) were purchased from Thermo Fisher, p17-specific Caspase 3 (25546-1-AP), Krt-14 (10143-1-AP) and Krt-18 (10830-1-AP) are purchased from ProteinTech.

### Examine of gene expression level

Bladder samples were snap frozen in liquid nitrogen, homogenized with a mortar and pestle, and RNA extracted with the TRIzol (Invitrogen). Quantitative RT-PCR and western blot was performed as previously described^39^,^40^. The primers used for RT-PCR is listed below. Antibodies of mouse E-cadherin (20874-1-AP), Vimentin (10366-1-AP), Snail1 (13099-1-AP), Snail2 (Slug, 12129-1-AP), Twist1 (18125-1-AP), Zeb1 (21544-1-AP) and beta-actin (60008-1-Ig) were provided by ProteinTech.

qRT-PCR analysis are:

E-cad F: 5′-CTCCAGTCATAGGGAGCTGTC-3′

E-cad R: 5′-TCTTCTGAGACCTGGGTACAC-3′

Vim F: 5′-TCCACACGCACCTACAGTCT-3′

Vim R: 5′-CCGAGGACCGGGTCACATA-3′

Snail1 F: 5′-CACACGCTGCCTTGTGTCT-3′

Snail1 R: 5′-GGTCAGCAAAAGCACGGTT-3′

Snail2 F: 5′-CAGCGAACTGGACACACACA-3′

Snail2 R: 5′-ATAGGGCTGTATGCTCCCGAG-3′

Twist1 F: 5′-GGACAAGCTGAGCAAGATTC-3′

Twist1 R: 5′-CGGAGAAGGCGTAGCTGAG-3′

Zeb1 F: 5′-ACTGCAAGAAACGGTTTTCCC-3′

Zeb1 R: 5′-GGCGAGGAACACTGAGATGT-3′

Gapdh F: 5′-TGGCCTTCCGTGTTCCTAC-3′

Gapdh R: 5′-GAGTTGCTGTTGAAGTCGCA-3′

### Statistics

Data are presented as the means ± standard deviation (S.D.) or standard error (S.E.). All of the statistical analyses were performed using Excel (Microsoft, Redmond, WA) or Prism (GraphPad Software Inc., La Jolla, CA). The two-tailed Student’s t-test, one-way analysis of variance was used to calculate statistical significance. A P-value of <0.05 was considered significant.

## Additional Information

**How to cite this article**: Liang, Y. *et al*. Conditional ablation of TGF-β signaling inhibits tumor progression and invasion in an induced mouse bladder cancer model. *Sci. Rep.* **6**, 29479; doi: 10.1038/srep29479 (2016).
